# Electrostatic melting in a single-molecule field-effect transistor with applications in genomic identification

**DOI:** 10.1038/ncomms15450

**Published:** 2017-05-18

**Authors:** Sefi Vernick, Scott M. Trocchia, Steven B. Warren, Erik F. Young, Delphine Bouilly, Ruben L. Gonzalez, Colin Nuckolls, Kenneth L. Shepard

**Affiliations:** 1 Department of Electrical Engineering, Columbia University, New York, New York 10027, USA; 2 Department of Chemistry, Columbia University, New York, New York 10027, USA

## Abstract

The study of biomolecular interactions at the single-molecule level holds great potential for both basic science and biotechnology applications. Single-molecule studies often rely on fluorescence-based reporting, with signal levels limited by photon emission from single optical reporters. The point-functionalized carbon nanotube transistor, known as the single-molecule field-effect transistor, is a bioelectronics alternative based on intrinsic molecular charge that offers significantly higher signal levels for detection. Such devices are effective for characterizing DNA hybridization kinetics and thermodynamics and enabling emerging applications in genomic identification. In this work, we show that hybridization kinetics can be directly controlled by electrostatic bias applied between the device and the surrounding electrolyte. We perform the first single-molecule experiments demonstrating the use of electrostatics to control molecular binding. Using bias as a proxy for temperature, we demonstrate the feasibility of detecting various concentrations of 20-nt target sequences from the Ebolavirus nucleoprotein gene in a constant-temperature environment.

## Abstract (web summary)

DNA hybridization of two single-strands to form a double-stranded helix is widely used for genomic identification applications. Here, Vernick *et al*. record duplex formation of 20-mer oligonucleotide using a single-molecule field-effect transistor, where DNA kinetics is affected by electrostatic bias.

The study of DNA hybridization kinetics and thermodynamics is foundational to many technologies central to genomic diagnostics. Single-molecule experiments, including fluorescence-based methods such as resonance energy transfer^1^,^2^,^3^ and force-based methods^4^ such as atomic force microscopy (AFM)^5^ and optical tweezers^6^, can be used to investigate the kinetics of association (hybridization) and dissociation (melting) of two single-stranded oligonucleotides at equilibrium^7^,^8^,^9^,^10^,^11^. While ensemble versions of such experiments are possible, single-molecule versions are much more informative^12^,^13^,^14^. The hybridization of two single-stranded oligonucleotides to form a double-stranded helix, for example, is widely analysed using a two-state model, in which formation of the double-stranded helix is driven by the favourable enthalpy of base-pairing and base-stacking interactions, and melting of the double-stranded helix is driven by the favourable configurational entropy of the single-stranded random coil.

Point-functionalized single-walled carbon nanotube (SWCNT) devices^15^ have emerged as an all-electronic, label-free, single-molecule detection platform. This single-molecule field-effect transistor (smFET) is characterized by a conductance that is sensitive to charges localized within a few Debye lengths of a point defect that is generated on the SWCNT sidewall^16^. This site of functionalization serves as the point of attachment of a SWCNT-tethered probe molecule under study^17^. Under a source–drain voltage bias of 100 mV, the current signal levels of a typical smFET are tens of nA. smFETs have been successfully used for the study of DNA hybridization and melting kinetics^15^, and DNA^18^ and protein^19^,^20^ conformational dynamics.

The smFET encodes information temporally; signal amplitudes can vary as long as there is sufficient signal-to-noise ratio (SNR) contrast between the conductance states of the system. In DNA hybridization and melting experiments, the temperature is kept near the melting temperature, *T*_m_, allowing repeated hybridization and melting of the target DNA molecule to the SWCNT-tethered probe DNA molecule^21^. The resulting conductance versus time trajectories are used to extract target DNA concentrations, hybridization rates (*k*_hyb_), melting rates (*k*_melt_), equilibrium constant (*K*_eq_) and free energy of hybridization (ΔG^0^)^15^.

Probe DNA sequences of lengths >15–20-mer are required for practical applications in genomic identification, but these lengths present challenges in smFET studies. In particular, *T*_m_ will often exceed 60 °C, reducing the SNR (by increasing low-frequency noise and lowering the current signal^22^) and reducing device reliability (since coefficient of thermal expansion mismatches and the effect of corrosive chemical reactions increases with increasing temperature). In addition, hybridization and melting kinetics become more complex with longer probes because of the formation of relatively stable intermediates.

When measured as a function of temperature and target DNA concentrations, we find that *k*_hyb_ exhibits non-Arrhenius behaviour that is largely unaffected by temperature (but, because it reflects a bimolecular process, is relatively sensitive to target DNA concentration), while *k*_melt_ exhibits Arrhenius-like behaviour that it is exponentially sensitive to temperature (but, because it reflects a unimolecular process, is relatively insensitive to target DNA concentration). This electrostatic modulation of hybridization and melting kinetics allows bias to act as a proxy for temperature, with electrostatic melting (e-melting) possible at a fixed temperature, alleviating the need to operate smFET devices at elevated temperatures or as a function of temperature changes. Previous ensemble studies sometimes refer to this bias dependence from charged surfaces as electrochemical melting^23^, although the effects equivalent to those observed here were electrostatic in nature and did not involve charge transfer. In addition, electrostatic repulsion between the surface of the SWCNT and the probe DNA target DNA hybrid destabilizes metastable misaligned intermediates, which tend to depress *k*_hyb_ at high target concentrations. E-melting also allows for straightforward identification of single-nucleotide polymorphisms (SNPs) in target detection through a characteristic effective melting potential, *E*_m_ (ref. 24).

## Results

### smFET device realization and characteristics

smFETs are based on the introduction of a point functionalization on an isolated SWCNT sidewall with, in our case, nano-confined covalent modification with a diazonium salt reagent^18^ (see Supplementary Note 1 and Supplementary Fig. 1). Following the isolation of individual SWCNTs, they are electrically contacted in a field-effect transistor configuration (see Methods) and thoroughly characterized (see Supplementary Note 2 and Supplementary Fig. 2). Post fabrication, unfunctionalized SWCNT devices are subsequently passivated with a 30-nm-thick e-beam-exposed polymethyl methacrylate (PMMA)-resist layer, leaving a 30-nm deep exposed ‘window' used to confine the covalent modification (see Supplementary Note 3 and Supplementary Fig. 3).

**Figure 1:**
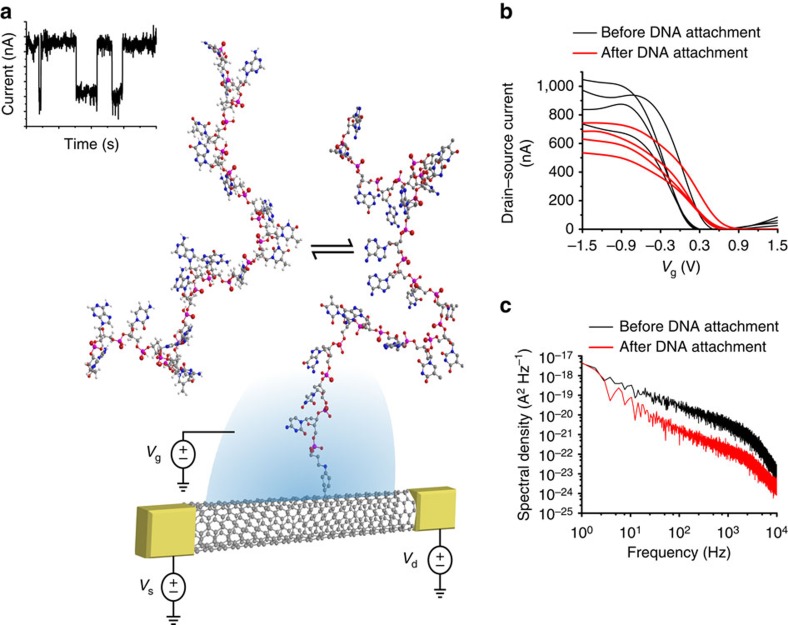
smFET device characteristics. (**a**) To-scale illustration of the smFET device structure. An amine-modified oligonucleotide probe is covalently coupled to a single defect site, generated on a single-walled carbon nanotube (SWCNT) sidewall via diazonium chemistry. The CNT acts as the channel of a field-effect transistor, transducing biomolecular charge into a conductance change in the smFET. Binding and melting reactions of a complementary oligonucleotide generate random telegraph signals (RTSs, shown in inset), which correspond to hybridization and melting events. Experiments are performed at a constant source–drain bias (*V*_s_) of 100 mV, while the electrolytic gate bias is modulated by an on-chip pseudo-reference electrode. (**b**) Representative transfer (*I*–*V*) characteristics of four CNT devices before and after point functionalization and DNA attachment. *V*_g_ is the bias on the platinum pseudo-reference electrode relative to the device. *V*_g_ is varied from −1.5 V to +1.5 V (in acetonitrile solution supported by 0.1 M tetrabutylamonium hexafluorophosphate). (**c**) The power spectral densities (PSDs) of the devices from **b** showing a strong flicker (1/*f*) noise component before (black line) and after (red line) modification. While the newly formed defect dominates transfer characteristics, the CNT sidewall becomes less sensitive to charge traps, leading to lower flicker noise.

A fabricated chip, consisting of 58 individual devices (see Supplementary Fig. 4), is stamped with a polydimethylsiloxane (PDMS) flow cell, placed directly above the isolated devices, which includes inlet and outlet ports for facile introduction of different reagents. A scaled representation of the smFET device with a tethered 20-mer probe sequence and a complementary target DNA is shown in Fig. 1a. The chip is mounted on a custom-made printed circuit board (see Supplementary Fig. 4) containing 58 independently addressable measurement channels that are simultaneously interrogated in real-time (Fig. 1a, inset). The diazonium-functionalized site in each device is conjugated to an amine-modified oligonucleotide probe. For our tethered probe DNA, we used an evolutionarily conserved sequence from the gene encoding the nucleoprotein of Zaire Ebolavirus: 5′-(Amino-C3)-CTGTGATTTCAAATTCAGTG-3′ (‘Amino-C3' denotes an amino modification positioned at the 5′ end through a three-carbon chain linker). The target DNA sequences studied here were composed of a fully complementary 20-mer (5′-CACTGAATTTGAAATCACAG-3′), a non-complementary 20-mer (5′ GTGATTTCACTTGCAATGTC-3′) and a 20-mer (5′ CACTGAATTTGAAATCACAC-3′) containing a SNP at the -3′ end relative to the fully complementary 20-mer. All experiments were performed in 43 mM phosphate buffer at pH 8.

Figure 1b shows the variation in drain current as a function of electrolytic gate bias (*V*_g_) at a fixed source-to-drain bias of 100 mV for four representative smFETs before and after diazonium and subsequent DNA point functionalization. *V*_g_ is established by applying a bias through the on-chip pseudo-reference platinum electrodes (fabrication and layout described in Methods and Supplementary Fig. 4). Reaction of diazonium with an *sp*^2^-hybridized carbon atom on the surface of the SWCNT results in a covalent bond and re-hybridization of the SWCNT surface carbon atom to *sp*^3^ (refs 25, 26), reducing the conductance, as shown in Fig. 1b. Before and after functionalization, the current noise spectrum is dominated by 1/*f* noise (flicker noise) as shown in the representative power spectral density of Fig. 1c. Following diazonium modification, the defect site dominates charge-carrier scattering rendering the rest of the SWCNT surface less sensitive to charge traps, as manifested in the reduced flicker noise. Before the introduction of the complementary 20-mer target DNA, one conductance level is evident. Upon the introduction of target DNA in solution, random telegraph noise-like transitions are observed between two discrete conductance levels, as shown in Fig. 1a, inset. These fluctuations are not observed in either a control experiment containing no target DNA or in a control experiment containing the non-complementary 20-mer target DNA (both presented in Supplementary Note 5 and Supplementary Fig. 5).

**Figure 2:**
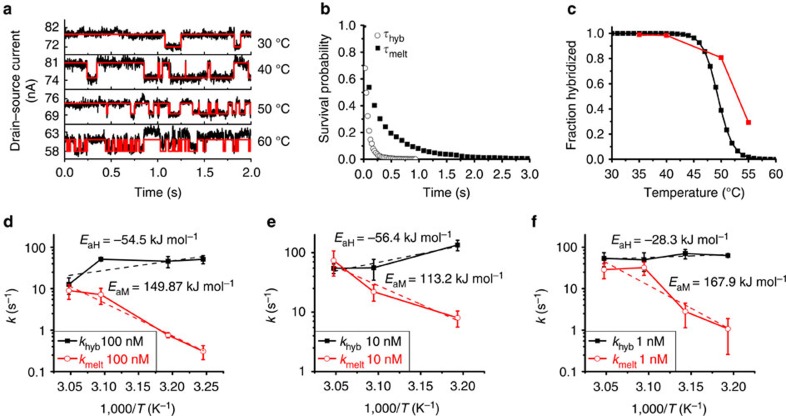
Temperature-dependent DNA hybridization and corresponding data analysis. (**a**) Time traces of 300-s length are recorded for complementary DNA target at a concentration of 100 nM. Overlaid raw real-time data (black) and idealized fits (red) for a temperature series from 30 to 60 °C. The rate of bi-stable activity increases with temperature. (**b**) A representative survival probability curve for the 50 °C trace from **a**, with an event count of 763. The fit quality is characterized by *R*^2^=0.999 and *R*^2^=0.998 for *τ*_low→high_ and *τ*_high→low_, respectively. (**c**) Comparison of nearest-neighbour calculated and single-molecule measured melting curves for a 100 nM complementary target showing the same temperature dependence as a ‘traditional' melting curve exhibiting a lower hybridized fraction with increasing temperature and a *T*_m_ of 52.99 °C compared with a calculated *T*_m_=49.66 °C. (**d**–**f**) Arrhenius plots for 100, 10 and 1 nM target concentrations, showing the temperature dependence of the melting and the hybridization rates. Error bars are calculated from five different 60-s intervals at each temperature.

The data are analysed by using an iterative event detection algorithm^27^. Conductance levels in each stochastic raw trajectory are assigned to discrete states, and an idealized trace is then drawn through the state assignments (as shown in Fig. 2a as a function of temperature). Plots of the survival probabilities in the hybridized or melted states (survival probability plots) are constructed from the idealized trajectories, yielding the rates of transition between the two conductance states (see Methods). Normalized, representative survival probability plots are shown in Fig. 2b for 100 nM complementary 20-mer target DNA at 50 °C, the melting temperature (*T*_m_) for this sequence, as predicted by nearest-neighbour model^21^ analysis (https://www.idtdna.com/calc/analyzer). Additional survival probability plots for 30, 40 and 60 °C are shown in Supplementary Fig. 6. The survival probabilities in the hybridized and melted states are best described by double-exponential functions with fast and slow time constants denoted as 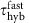 and 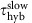, respectively, for the hybridized state and 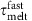 and 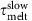, respectively, for the melted state. Each rate constant, *k*_melt_ and *k*_hyb_, is calculated as the reciprocal of its corresponding time constant.

For hybridization, we interpret the two components of the double-exponential functions as reflecting competing bulk (three-dimensional (3D)) and surface-based (one-dimensional (1D)) diffusion processes. Because the rates of association corresponding to 3D and 1D diffusion processes are expected to have different ligand concentration dependencies, whether 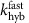 or 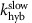 corresponds to the 3D or 1D diffusion processes will depend on target DNA concentration. At low target DNA concentrations (for example, 1 and 10 nM), we interpret 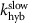
as corresponding to the 3D diffusion process, since the 1D diffusion process is expected to be faster at lower target DNA concentrations. Above a certain target DNA concentration, however, the 3D diffusion process is expected to be faster than the 1D diffusion process (1D diffusion coefficients are inversely proportional to ligand concentrations^28^,^29^,^30^), and we interpret 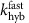 as corresponding to the 3D diffusion process at 100 nM. Hereafter, *k*_hyb_ is chosen as 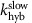 or 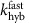 as a function of target concentration to always represent the 3D diffusion process. The melting process, which is also described by a double-exponential function, should not depend on target concentration, which is achieved by choosing the slow and fast melting rates for *k*_melt_ as a function of concentration in the same manner as done for *k*_hyb_. Physically, this is a result of the fact that surface-mode melting is slower at higher target concentrations, due to the higher concentrations of surface-adsorbed negatively charged target (see Supplementary Note 7 and Supplementary Fig. 7).

### Single-molecule kinetics of 20-mer oligonucleotide hybridization

Single-molecule conductance trajectories obtained for the hybridization and melting of the tethered probe DNA in the presence of 100 nM of complementary 20-mer target DNA at various temperatures are shown in Fig. 2a. The higher-conductance state dominates at 30 °C, but this gradually shifts as a function of increasing temperature, such that the lower-conductance state dominates at 60 °C (the same behaviour is observed over a longer time period; see Supplementary Note 8 and Supplementary Fig. 8). From the corresponding survival probability plots, shown in Fig. 2b, *k*_hyb_=15.50 s^−1^, while *k*_melt_=15.52 s^−1^. Plots of the fraction of probe DNA that is hybridized as a function of temperature (that is, melting curves) for each target DNA concentration can be calculated from 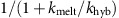. Figure 2c shows that the experimentally determined melting curve at 100-nM target DNA concentration (red squares and trend line), which results in a *T*_m_ of 52.99 °C, compares favourably with a predicted melting curve with a *T*_m_ of 49.66 °C, obtained using a nearest-neighbour calculation (https://www.idtdna.com/calc/analyzer).

As shown in Fig. 2d, the temperature dependence of *k*_hyb_ and *k*_melt_ exhibits Arrhenius-like behaviour, with apparent activation energies *E*_aM_ and *E*_aH_ of 149.87 (±19.33) kJ mol^−1^ and −54.53 (±44.76) kJ mol^−1^, respectively. As expected, *k*_melt_ shows a strong Arrhenius-like temperature dependence, while *k*_hyb_ has a weak anti-Arrhenius behaviour, reflecting the reduced enthalpy of hydrogen bonding at elevated temperatures. At different target DNA concentrations (Fig. 2e,f), the hybridization and melting kinetics follow a similar temperature dependence.

The target DNA concentration dependence of *k*_hyb_ is presented in Supplementary Fig. 7b. A relatively linear target concentration dependence is observed, as expected from the diffusion-limited process. At a target concentration of 100 nM, however, *k*_hyb_ does not appreciably increase because under these conditions, *k*_hyb_ is reaction-limited, an observation consistent with ensemble^31^,^32^ and single-molecule fluorescence resonance energy transfer studies^2^. In addition, *k*_hyb_ at the 1 and 10 nM concentrations are expected to be accelerated compared to the 100 nM concentration, since the contribution of intermediate pathways to *k*_hyb_ becomes insignificant at these lower concentrations, again consistent with ensemble studies^13^,^33^,^34^.

### The effect of electrostatic bias on hybridization kinetics

Temperature is often an inconvenient means to modulate *k*_hyb_ and *k*_melt_ in smFET-based genomic diagnostics, both because of the high temperatures that may be required to melt longer oligonucleotides and because it may not be possible to control or change temperature in large device arrays. The smFET device structure, however, provides the unique capability to modulate *k*_hyb_ and *k*_melt_ using an electrostatic bias between the device and the surrounding electrolyte. At an applied bias of *V*_g_=0 V, the CNT is not at zero charge and the reaction cannot be assumed to be solely thermally modulated. This bias point is, however, a reasonable reference point from which any additional charge would provide sufficient energy to modulate the kinetics. We find that *k*_melt_ increases when the smFET device is placed at a negative potential relative to the surrounding electrolyte. While studies of DNA hybridization and melting kinetics (and genomic assays) generally rely on either thermal or chemical (stringency washing) methods to modulate hybridization and melting, electrostatically driven modulation of hybridization and melting, conceived 40 years ago on macro-electrodes at the ensemble level^35^,^36^,^37^,^38^, has seen relatively little study and no single-molecule characterization.

**Figure 3:**
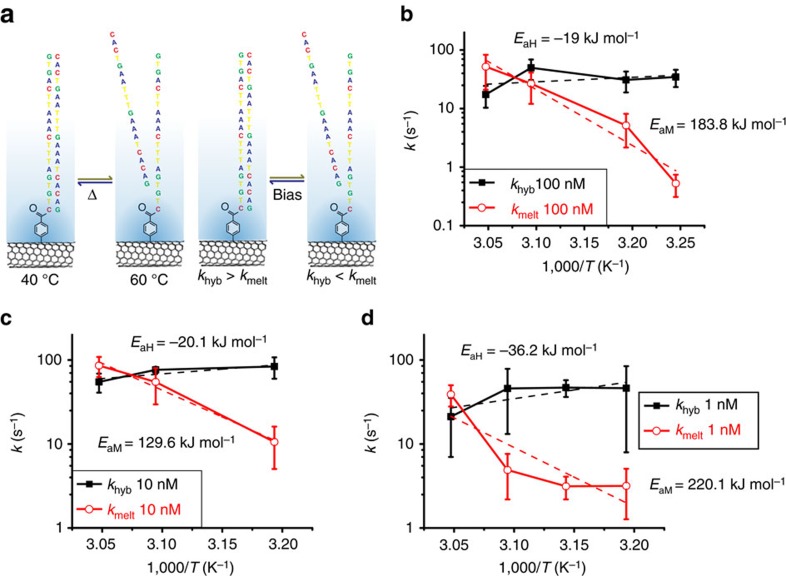
The effects of bias on hybridization and melting kinetics. (**a**) Comparison of temperature and bias-dependent melting. With increasing temperature, the melting rate increases and hybridization rate decreases. Equivalently, when the gate bias increases, the equilibrium favours the melted state. (**b**–**d**) Arrhenius plots showing how application of gate bias affects the kinetics of melting and hybridization for 100, 10 and 1 nM complementary target. Upon the application of 300 mV of gate bias, the entropy of activation for the melting reactions increases, while the enthalpy of activation for the hybridization reaction decreases. Error bars are calculated from five different 60-s intervals at each temperature.

The electrostatic force generated by the application of 300 mV provides sufficient energy to alter *k*_hyb_ and *k*_melt_ compared to those observed at a *V*_g_ of 0 V. As shown in Fig. 3a, at a negative *V*_g_, electrostatic repulsion of the negatively charged nucleotides within a Debye sphere around the SWCNT surface decreases *k*_hyb_ and increases *k*_melt_. (We have previously modelled this Debye screening effect in the smFET platform^17^). The temperature dependence of *k*_hyb_ and *k*_melt_ obtained under a 300-mV gate bias is shown in Fig. 3b–d using the complementary 20-mer oligonucleotide target DNA at different target DNA concentrations.

The effect of *V*_g_ on *k*_hyb_ and *k*_melt_ can be analysed using transition-state theory^39^. Specifically, *k*_hyb_ and *k*_melt_ as a function of temperature at a particular *V*_g_ can be fit to an equation of the form: 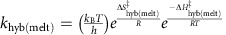, where 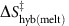 is the activation entropy for hybridization (melting), 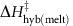 is the activation enthalpy for hybridization (melting), *k*_B_ is Boltzmann's constant, *R* is the gas constant, *h* is Planck's constant and ‡ denotes the transition state. Although *V*_g_ affects both *k*_hyb_ and *k*_melt_, the strongest *V*_g_ dependence occurs for *k*_melt_. By comparing the Arrhenius analyses of *k*_melt_ presented in Figs 2d–f and 3b–d, one finds that 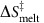 increases with the application of 300-mV bias by 115.03 (±62.37), 58.40 (±41.94) and 157.47 (±97.7) J K^−1^ mol^−1^ for 100, 10 and 1 nM target concentrations, respectively. This increase in 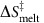 suggests that a disordered transition state drives a faster reaction once electrostatic bias is applied.

At the same time, the activation enthalpies of hybridization 
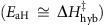
 become more positive with the application of bias. 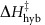 increases by 35.5, 36.3 and 7.9 kJ mol^−1^ for 100, 10 and 1 nM target concentrations, respectively, revealing the increased enthalpic penalty of hybridization under 300 mV bias. This is the result of a repulsive electrostatic force that promotes melting and inhibits hybridization, eliminating the contribution of intermediate pathways to *k*_hyb_ in the absence of bias. Interestingly, we find that 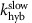 at a target DNA concentration of 100 nM is strongly affected by the application of an electrostatic bias, whereas *k*_hyb_ at other target DNA concentrations are not (Supplementary Fig. 7c), suggesting that the application of electrostatic bias increases the rate of the 1D diffusion process at the 100 nM target DNA concentration. This observation supports the hypothesis that the surface-based (1D) hybridization at high target DNA concentrations is decelerated due to steric interferences from other adsorbed 20-mer (see Supplementary Note 7). In the presence of electrostatic bias, this steric hindrance is mitigated by a bias that repels adsorbed DNA.

### Using electrostatic bias as a proxy for temperature

**Figure 4:**
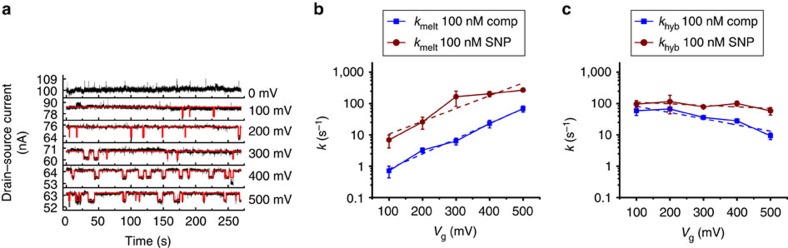
Sequence dependence and the effect of e-melting on reaction kinetics. (**a**) Overlay of raw real-time data (black) and idealized fits (red) for a bias series ranging from *V*_g_=0 V to *V*_g_=500 mV of a 100 nM complementary target. At 0 V, no melting events are observed and the hybridized state is dominant at 40 °C. When *V*_*g*_ is increased, the melting rate increases, demonstrating longer and more frequent melting events. (**b**) *k*_melt_*s* of a complementary target DNA at a temperature of 40 °C increases exponentially with increasing *V*_g_. Behaviour of a target containing a single-base mismatch (SNP) has a noticeably smaller activation energy and higher melting rate constant at each bias point. (**c**) *k*_hyb_ is less sensitive to bias. At higher bias values, *k*_hyb_ decreases, indicating that base-pairing is affected under repulsive electrostatic force, while the SNP, which cannot pair its terminal base, does not show this effect. Error bars are calculated from five different 60-s intervals at each temperature.

The effects of *V*_g_ on the kinetics and thermodynamics of DNA hybridization and melting suggest that electrostatic modulation may be used to study hybridization and melting kinetics and thermodynamics at a fixed temperature by sweeping *V*_g_ instead of temperature. Figure 4a shows raw conductance trajectories and idealized hybridization and melting trajectories for one measured smFET device at a fixed temperature (40 °C), over a range of *V*_g_. At a *V*_g_ of 0 V, only one conductance state, that associated with the hybridized state of the probe DNA, is apparent. With increased electrostatic bias, however, a low-conductance state associated with the melted state of the probe DNA is observed. Current histograms for each bias point are shown in Supplementary Fig. 9. Two-state transitions were not observed with the non-complementary, 20-mer target DNA (as shown by the control conductance trajectories presented in Supplementary Fig. 10).

Figure 4b,c shows plots of *k*_melt_ and *k*_hyb_ as a function of *V*_g_ for both the fully complementary 20-mer target DNA and SNP-containing 20-mer target DNA at a temperature of 40 °C and 100 nM target DNA concentration. These plots show electrostatically modulated kinetics, which bears a striking resemblance to thermally modulated kinetics. We can model this bias dependence^40^,^41^ in 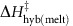 by 
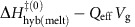
, where 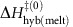 is the hybridization (melting) activation enthalpy in the absence of bias and *Q*_eff_ is the effective molecular charge on the target interacting with the applied field. The product of *Q*_eff_*V*_g_ is the reduction in the reaction energy barrier. In particular, for *k*_melt_ of the fully complementary 20-mer target DNA, 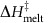
=3.57 (±0.60) kJ mol^−1^ for a voltage-driven reaction, whereas 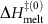
=149.87 (±19.33) kJ mol^−1^ in absence of bias. *k*_melt_ is larger for the SNP-containing 20-mer target DNA, as expected. In particular, 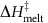 for the fully complementary 20-mer target DNA is larger compared to the SNP-containing 20-mer target DNA (3.57 (±0.60) kJ mol^−1^ compared to −2.81 (±1.84) kJ mol^−1^, respectively), a remarkable sensitivity for detecting a SNP. *k*_hyb_ is less sensitive to electrostatic bias, as shown in Fig. 4c, providing a relationship similar to the temperature dependence of Fig. 2d. Interestingly, at higher bias values, *k*_hyb_ for the fully complementary 20-mer target DNA decreases, indicating that pairing of the terminal base is highly sensitive to bias, whereas in the SNP-containing 20-mer target DNA, which lacks the ability to fully base pair, no such sensitivity is observed.

**Figure 5:**
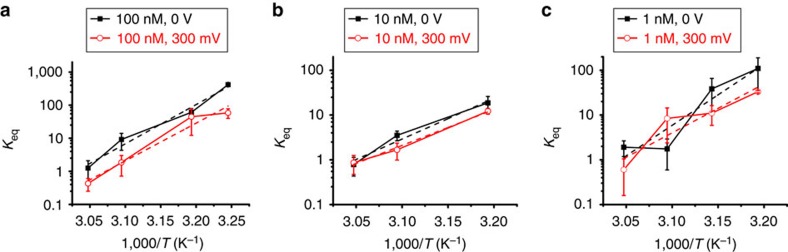
Reaction thermodynamic parameters are affected by electrostatic force. Van't Hoff plots depict the effect of bias on the free energy landscape, revealing an order of magnitude lower equilibrium constants and higher enthalpy for the 300 mV biased reaction at (**a**) 100 nM complementary target, where an increase in Δ*H°* of 6.5 kJ mol^−1^ making hybridization less favourable, (**b**) at 10 nM with an enthalpy increase of 38.46 kJ mol^−1^ and (**c**) at 1 nM with an enthalpy increase of 74.78 kJ mol^−1^. Error bars are calculated from five different 60-s intervals at each temperature.

The Van't Hoff expression for the equilibrium constant (*K*_eq_) governs the DNA hybridization reaction, determines the thermodynamics of the equilibrium state and is given by: 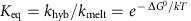, where 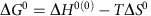, where 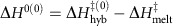 is the enthalpy in the absence of electrostatic bias and 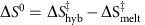. The dependence of *K*_eq_ on temperature is shown for the fully complementary 20-mer target DNA at a concentration of 100 nM in Fig. 5a with and without the application of 300 mV of *V*_g_. In the absence of electrostatic bias, an enthalpically favoured process with Δ*H°*=−211.61 (±15.13) kJ mol^−1^ drives hybridization until the entropic cost becomes sufficiently high (at temperatures above 52 °C), as shown in Fig. 5a. The Van't Hoff plots and enthalpies obtained for 10 and 1 nM concentrations (shown in Fig. 5b,c, respectively) also reveal large and negative enthalpies Δ*H°*=−178.50 (±17.58) kJ mol^−1^ for 10 nM and Δ*H°*=−231.19 (±44.97) kJ mol^−1^ for 1 nM without electrostatic bias.

The effect of electrostatic modulation of *K*_eq_ is clearly observed in Fig. 5a–c and results from the bias dependence of the enthalpy, 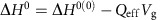. When the bias is raised to 300 mV, Δ*H°* increases to −205.11 (±3.66) kJ mol^−1^ for the 100 nM target DNA concentration, due to less favourable base-pairing, reducing *K*_eq_ by an order of magnitude. The reaction enthalpy is also increased at the lower target DNA concentrations (Fig. 5b,c), with Δ*H°*=−140.04 (±6.92) kJ mol^−1^ and Δ*H°*=−156.41 (±76.42) kJ mol^−1^ for the 10 and 1 nM target DNA concentrations, respectively.

**Figure 6:**
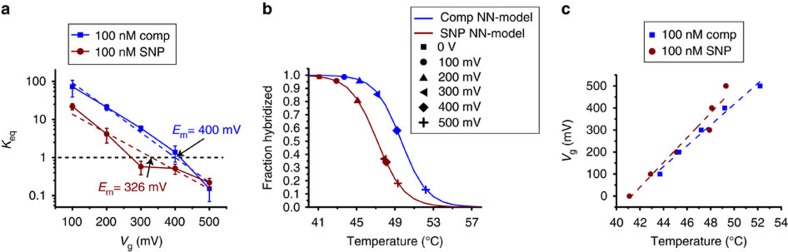
Sequence-dependent effects of electrostatic force. (**a**) Dependence of *K*_eq_ on *V*_g_, demonstrating higher values for the complementary compared to the SNP across all *V*_g_ values. *K*_eq_=1 defines a melting potential of *E*_m_=400.05 (±2.55) mV for the complementary target and *E*_m_=326.45 (±1.33) mV for the SNP; *R*^2^ values for the SNP and comp are 0.95 and 0.97, respectively. Error bars are calculated from five different 60-s intervals at each bias value. (**b**) An effective *T*_m_ curve showing the extrapolated temperatures for each hybridized fraction of both complementary and SNP target against their perspective nearest-neighbour models; and (**c**) calibration curves demonstrate the interchangeable effect of bias and temperature on reaction rates and further correlate each bias point with an effective temperature.

An Arrhenius-like analysis of the plots of *K*_eq_ as a function of *V*_g_ allows one to easily extract *Q*_eff_ and Δ*G°.* Changing the 5′ terminal base from a guanine to a cytosine in the SNP-containing target DNA relative to the fully complementary DNA target is the most destabilizing mismatch in terms of enthalpy^42^, reducing the *T*_m_ by ∼2.6 °C. A similar change is observed in Fig. 6a, for the bias dependence of *K*_eq_. We can define a melting potential (*E*_m_) as the potential at which *K*_eq_=1. *E*_m_ is 400.05 (±2.53) mV for the fully complementary target DNA, but only 326.45 (±1.33) mV for the SNP-containing target DNA. ΔG° is −15.87 (±1.03) kJ mol^−1^ for the fully complementary target DNA and −9.16 (±1.37) kJ mol^−1^ for the SNP-containing target DNA, reflecting the fact that the hybridization reaction is more thermodynamically favourable for the fully complementary target DNA. The effective charge (*Q*_eff_) was previously estimated to be 0.1q per nucleotide^43^. *Q*_eff_ associated with hybridization is slightly higher for the fully complementary target DNA in comparison with the SNP-containing target DNA (0.40q compared to 0.28q or ∼4 nt compared to ∼2.87 nt), consistent with the existence of an additional, negatively charged nucleotide closer to the surface of the SWCNT in the fully complementary target DNA case. In all these experiments, controls employing the non-complementary 20-mer target DNAs fail to produce any two-state activity (see Supplementary Fig. 10).

Figure 6b correlates the bias-dependent melting at a fixed temperature of 40 °C to temperature-dependent melting. From a plot of the fraction of probe DNA that is hybridized versus *V*_g_, we extract an effective *T*_m_, where the fraction hybridized is 0.5, of 49.5 °C for the fully complementary target DNA and 47.1 °C for the SNP-containing target DNA. The difference between these two values (2.45 °C) is in good agreement with the expected difference in *T*_m_ (2.6 °C). With electronic control, we can influence the kinetics and thermodynamics of DNA hybridization and melting, and provide a sequence-dependent calibration between applied bias and temperature as shown in Fig. 6c. One of the mechanisms for this bias dependence may be destabilization of base-stacking, as previously hypothesized in ensemble studies^44^. Our results support this explanation by observing increased activation enthalpy for hybridization upon application of negative bias.

## Discussion

It is interesting to contrast these studies with surface-based ensemble assays based on hybridization, in which the number of bound target within a given area on a probe surface, usually determined fluorescently, constitutes the signal. Target concentrations, affinities and non-specific adsorption collectively affect this signal and extensive optimization is necessary to separate these factors^45^,^46^,^47^. In the single-molecule assays proposed here, however, all information is encoded temporally in the binding and unbinding kinetics^48^, leaving the system relatively immune to variability in signal amplitudes as long as SNR performance is maintained. smFET arrays have the same multiplexing potential as DNA microarrays but do not require any target amplification through PCR. Avoiding these the labour-intensive preparation steps required for PCR further simplifies potential analysis with label-free smFET-based single-molecule assays.

In summary, we have developed an all-electronic approach for measuring the kinetics and thermodynamics of DNA hybridization and melting as a function of temperature and electrostatic bias applied through the surrounding electrolyte. We have demonstrated that, with electronic control, we can influence the kinetics and thermodynamics of DNA hybridization and melting in a manner equivalent to temperature. An smFET array was utilized to transiently sense single-base-pair differences of two different 20-mer target DNA sequences—a fully complementary target DNA and a SNP-containing target DNA that are both derived from a gene encoded by the Zaire Ebolavirus. These studies pave the way for further work in the application of smFETs to genomic identification in which bias can be used as a proxy for temperature.

## Methods

### Device fabrication

Carbon nanotube smFET devices are constructed in the following manner. First, nanotubes are grown at 890° C on the surface of 1 × 1 cm^2^ bare Si (500 μm)/SiO_2_ (285 nm) die via chemical vapour deposition. The average spacing between grown nanotubes is ∼1 nanotube per 100 μm. Second, 64 source and drain electrodes (each 8 mm × 15 μm, segmented into 16 blocks of four pairs) are patterned orthogonal to the growth direction of nanotubes using a bilayer-resist photolithography process. The gap between electrodes is 4 μm, defining the nanotube channel length.

Titanium metal (100 nm) is deposited via electron-beam deposition, and the photoresist stack is lifted off. Large rectangular bars (8 mm × 100 μm) are photolithographically defined above and below the electrode pattern, and e-beam platinum (100 nm) is deposited to act as a pseudo-reference gate electrode. Following SEM inspection, nanotubes that bridge source–drain electrode pairs are identified. Those that transit the electrode gaps and are likely single-walled (diameter <2 nm, as confirmed via Raman spectroscopy and AFM characterization) are protected with a photoresist mask. All other nanotubes are etched with oxygen plasma in a Technics RIE tool (250 mtorr O_2_, 50 W, 12 s).

Subsequently, nanowells are patterned in a thin electron-beam resist (950 K PMMA A2), in a similar manner to a formerly described method^18^. The resist is diluted by 25% before use to achieve a nominal layer thickness of ∼30 nm. Then, it is spun at a spin speed of 8,000 r.p.m. for 1 min. The sample is baked on a hotplate at 180 °C for 2 min and loaded into a high-resolution e-beam lithography writer (Nanobeam nB4). Nanowells measuring 24 μm long by 30 nm wide are written using 0.5-nA beam current, thereby producing a 1:1 aspect ratio. The pattern is developed for 100 s in a cold mixture of 3:1 IPA:DI H_2_O (4 °C), and the width of nanowells is assessed via AFM.

### Diazonium functionalization

Devices are exposed to 10 mM 4-formylbenzene diazonium hexafluorophosphate dissolved in 100 mM sodium phosphate buffer solution with pH 8.0 overnight on a shaking tray and in the dark. Afterwards, the thin PMMA layer is removed in heated acetone (55° C) for 2 h, rendering the surface of the chip clean again. Chips are wirebonded to ball-grid array packages using an automated wirebonder, and subsequently placed onto a custom-made circuit board described in detail below.

### DNA attachment

After the functionalization stage, and after being mounted onto the circuit board, wirebonded chips are exposed to 10 μM of probe DNA (Zaire Ebolavirus nucleoprotein gene, position 1,158: 5′-CTGTGATTTCAAATTCAGTG-3′) in a 100 mM sodium phosphate buffer solution with pH 8.0, with added 200 μM sodium cyanoborohydride (NaBH_3_CN) dissolved in 1 N NaOH, which is used to reduce the Schiff base formed between the amine and aldehyde, converting into a stable secondary amine.

### Measurement set-up

The measurement set-up consists of a PDMS microfluidic channel for interfacing solution with the fully fabricated smFET devices, a custom-made printed circuit board for data acquisition, and a temperature sensor/controller for fixing and modulating the temperature in the vicinity of the chip surface.

The circuit board contains 58 independently addressable measurement channels that are simultaneously interrogated in real-time. The circuitry for each channel incorporates tunable drain and source potentials, and is composed of two mutable gain stages: a front-end transimpedance amplification stage with a fixed resistive gain of 1 MΩ, followed by an inverting voltage amplifier with variable gain from 2 × to 200 × . Each channel, furthermore, utilizes a second-order active filter topology, limiting the signal bandwidth to 5 kHz. Readings from each channel are sampled at a rate of 25 kSps. The hardware–software interface is governed by an Opal Kelly XEM6010 FPGA module, which connects to multiplexers and analog-to-digital converters on the printed circuit board, and with the PC via a USB 2.0 connection.

The PDMS microfluidic mould is constructed from a pattern drawn on a thick SU-8 layer. Such microfluidic channels have the following dimensions: 7-mm long, 750-μm wide and roughly 500-μm tall. Inlet and outlet holes are punched into the channel, and two sterile tubing segments are inserted. A syringe pump connected to the outlet terminal withdraws fluid exiting the channel, thus allowing full control over flow rates.

Temperature control is achieved by using a commercially available Thermostream unit capable of monitoring and modulating the temperature of forced air within a manufactured enclosure surrounding the fabricated chip and microfluidics. The temperature is allowed to reach steady state before an experimental condition is recorded.

### Data analysis

Once acquired through the FPGA-to-PC interface, data are post-processed using customizable MATLAB scripts. Local drift for 5 min of transient recording from each measurement channel is systematically removed. Resulting signals are low-pass filtered with a fourth-order Butterworth filter to 1 kHz to eliminate noise close to the cutoff frequency of the anti-aliasing filter of each channel. Every trace is further analysed using an iterative event detection algorithm^27^, which assumes a two-state model, with wandering baseline correction. Traditionally, this single-molecule data analysis methodology is applied to evaluate current blockades due to nanopores, but the same technique can be extended to any signature with two-state random telegraph noise.

Compared to an alternate signal processing paradigm for single-molecule trajectories, the hidden Markov model, the iterative detection algorithm utilizes rudimentary statistical metrics (for example, moving average, RMS noise level) rather than Markovian matrices and machine learning principles^49^. Consequently, the execution speed is faster, allowing for more rapid tuning of parameters by the user. Idealized traces, resulting from fits to the raw data in the iterative detection algorithm, are used to extract single-molecule binding kinetics information. Assuming the same two-state model as before, events are classified into a ‘low'- and a ‘high'-conductance state. Each idealized data trace for a given experimental condition is divided into five equal parts, from which cumulative density functions are constructed for each state. Each cumulative density function is normalized to the number of event counts, thus yielding survival probability plots. Average kinetic DNA hybridization/melting rates and associated error bars are calculated from them. Algorithms for this portion of the analysis are adapted from HaMMy scripts previously written in MATLAB^50^.

Errors for the derived kinetic and thermodynamic parameters were calculated from the s.e.'s of the corresponding least-square weighted fits. The errors for the derived differences between thermodynamic values were combined in quadrature. The errors for parameters derived from quotients (that is, the *E*_m_, calculated by dividing the intercept by the slope) were obtained by taking the root of the sum of the squares of the fractional errors in the original quantities.

Signal specificity was further demonstrated by analysis of SNR of a two-state signature of a fully complementary strand. The dependence of SNR on gate bias, as shown in Supplementary Fig. 11, reveals an increasing trend, indicating that bias-modulated performance of the device was not responsible for the obtained signals.

### Data availability

All data supporting the findings of this study are available within the article and its Supplementary Information files or from the corresponding author upon reasonable request.

## Additional information

**How to cite this article:** Vernick, S. *et al*. Electrostatic melting in a single-molecule field-effect transistor with applications in genomic identification. *Nat. Commun.* **8,** 15450 doi: 10.1038/ncomms15450 (2017).

**Publisher's note:** Springer Nature remains neutral with regard to jurisdictional claims in published maps and institutional affiliations.

## Supplementary Material

Supplementary Information Supplementary Figures, Supplementary Notes and Supplementary References
