# Supplementary material for: Electrostatic melting in a single-molecule field-effect transistor with applications in genomic identification
Source: Nat Commun. 2017 May 18;8:15450. doi: 10.1038/ncomms15450 (PMC5454367; doi:10.1038/ncomms15450)
Supplement: Supplementary Information — Supplementary Figures, Supplementary Notes and Supplementary References [file ncomms15450-s1.pdf]

Supplementary Note 1

FBDP characterization

Reactions of SWCNT with aryl diazonium salts have been widely studied for covalent functionalization of nanotubes[1, 2, 3, 4]. The reaction of the diazonium salt with the SWCNT proceeds through a two-step process -- selective adsorption, mediated by electron transfer from the nanotube to the diazonium salt, followed by a covalent reaction which results in localization of electrons and formation of an impurity state at the Fermi level[5]. We use the diazonium reagent formylbenzenediazonium hexafluorophosphate (FBDP) for this study because of its stability and compatibility with[6, 7] well-established bio-conjugation methods[8].

Formylbenzenediazonium hexafluorophosphate (FBDP) was synthesized according to established protocols[6] and was further characterized by FTIR (Perkin Elmer) as shown in Supplementary Figure 1a. A typical aldehyde-group IR mode at ~1710cm^{-1} is observed. Additionally, the reaction of FBDP with carbon nanotubes (CNTs) was characterized by XPS (Phi 5500), following a CNT modification reaction and a succeeding amine coupling to the aldehyde. The C1s spectra shown in Supplementary Figure 1b (with a corresponding curve fit) was collected following incubation with 10 mM FBDP (dissolved in acetonitrile, reacted for 2 hours with CVD-grown carbon nanotubes on a Si/SiO_2 chip). The nanotube C-C bond peak at 285.1 eV is observed. Resolution is insufficient to determine the composition of sp^2 versus sp^3 carbon in this peak. A wider peak at a binding energy of 287.6 eV is visible, a carbonyl peak that may arise from the aldehyde group of FBDP. Post-amination (with di-amino butane) this peak (at 287.1 eV) becomes slightly broader and less intense and could be related to an amide (N-C=O) or to a carbonyl (C=O). Also, while scanning the N1s range, a low intensity peak is now clearly visible, indicating the presence of an amine, as seen in Supplementary Figure 1c.

Additionally, electrochemical characterizations were performed and some examples are given in Figure S1d. Cyclic voltammetry measurements (CHI 760D potentiostat, employing an electrochemical cell with Au working electrode, Ag/AgCl reference electrode and Pt-wire counter electrode all from BASi) are performed with FBDP diluted in either organic or aqueous solvent, revealing an irreversible electro-reduction of FBDP at low anodic potentials and demonstrating the chemical stability of the N_2 leaving

group. At a potential of 200 mV vs. Ag/AgCl, FBDP is reduced and grafts to the working electrode, completely fouling the surface. After three CV cycles, no electrochemical activity can be recorded with the working electrode, as demonstrated with the redox couple $[Fe(CN)_6]^{3-}/[Fe(CN)_6]^4$ and with ferrocene (data not shown).

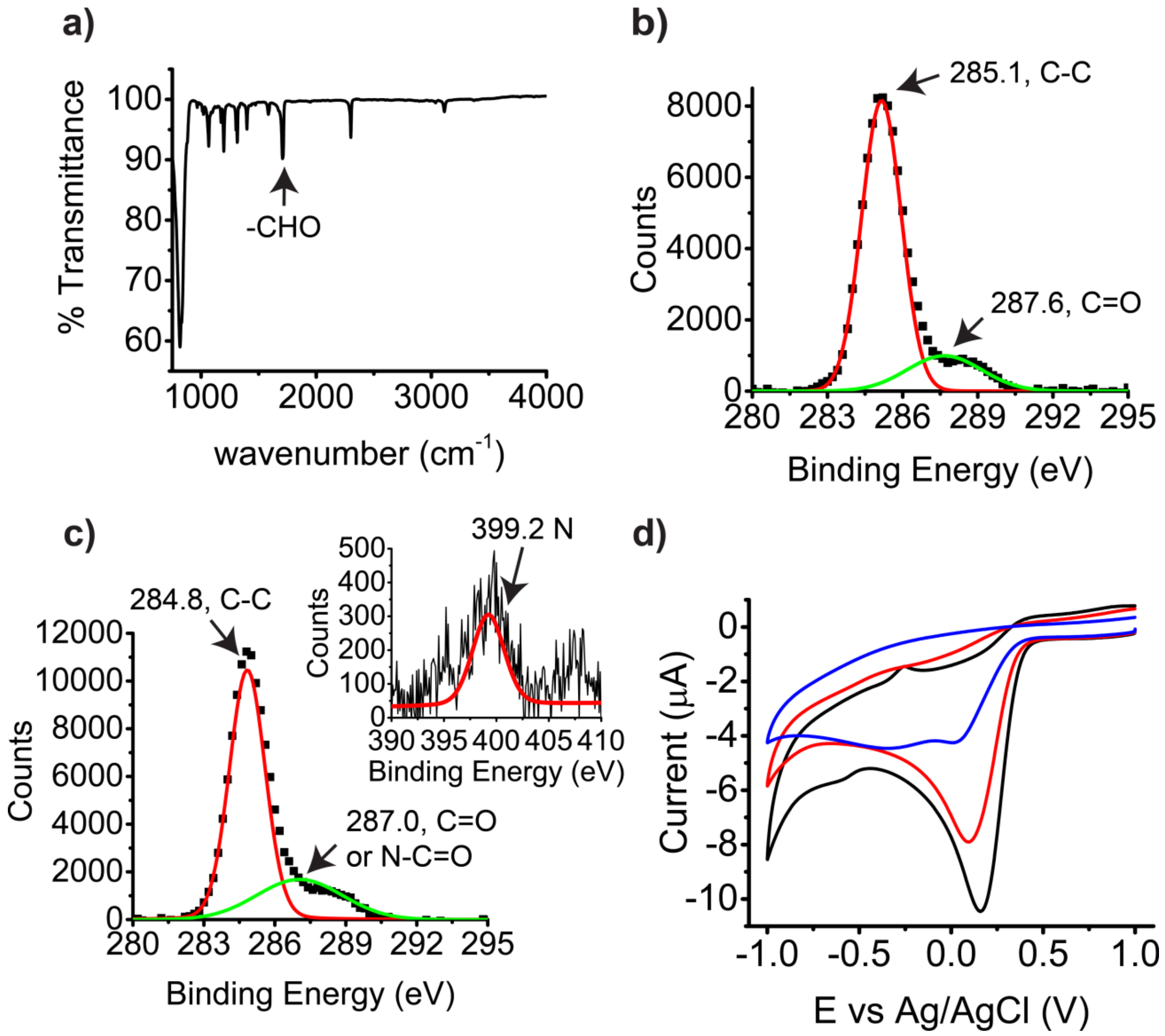

Supplementary Figure 1. Various characterization assays for FBDP and its reaction with CNTs. a) FTIR of the synthesized reagent revealing the IR vibrational mode at ~1707 cm^{-1}. b) XPS C1s spectra of FBDP reacted CVD-grown CNTs on Si/SiO2. Peak at 285.1 eV indicating the C-C sp^2-sp^3 structure and a carbonyl peak at 287.6 eV possibly related to the added aldehyde group. c) Post amination this peak is slightly broader, which may indicate the presence of amide. Inset shows the N1s spectra collected following amination, with an amine peak at ~400 eV. d) Cyclic voltammetry (+1V to -1V at a scan rate of 500mV/sec in Acetonitrile supplemented with 0.1 M Tetrabutylammonium hexafluorophosphate electrolyte) showing the irreversible electro-reduction of FBDP on a gold working electrode at Ep=+200mV resulting in complete passivation of the electrode surface.

Supplementary Note 2

Device Characteristics (I-V curves, AFM and SEM images)

Devices were thoroughly characterized prior to use by a combination of electron microscopy, AFM, electronic (I-V) and chemical (XPS, Raman) analysis. First, CVD-grown chips (from metal catalyst) are scanned by an electron microscope (Zeiss) in order to identify a CNT for subsequent isolation. A typical CNT is shown in SEM micrograph at Supplementary Figure 2a. Typically, a CVD-grown chip contains a few nanotubes that span > 5mm, allowing a large number of devices to be fabricated from a single CNT. Before isolation of a CNT a resonant Raman spectroscopy analysis (Renishaw in-via confocal Raman microscope using either 532nm or 630nm laser) is performed. The ratio of the D band ("disorder mode") to the tangential G band is measured, which serves as an indicator to the number of intrinsic CNT defects. In addition, the electronic structure is elucidated by analyzing the G mode components. The radial breathing mode (RBM) is collected, as well, indicating the CNT diameter[9]. Only CNTs demonstrating no scattering at the D band (apparent "pristine" nanotubes) are isolated for device fabrication. Furthermore, double-walled carbon nanotubes, detected infrequently by their RBM signature, are discarded. Typical Raman spectra of a CNT grown on Si/SiO_2 is shown in Supplementary Figure 2c. An AFM measurement, confirming the CNT diameter is performed by a Brucker AFM in tapping mode, as shown in Supplementary Figure 2b. Following initial characterization, the CNT is isolated into single devices by using photolithography and oxygen plasma etching (250mTorr O_2 at 50 Watt, 15 sec). Post-isolation, the chip is SEM-scanned again, verifying the isolated individual CNT devices, as shown in Supplementary Figure 2d. Finally, the individual devices' I-V characteristics are measured by backgating in air (-10V to+10V at a fixed source-drain bias of 100mV) as shown in Supplementary Figure 2e. The transport characteristics in all of these current-versus-V_g curves reveal high current levels at negative V_g while the n-type conductance at positive V_g is significantly lower, a result that is typical of semiconducting nanotubes because the work function of the contact metal gives rise to a high series contact resistance for the n-branch[10].

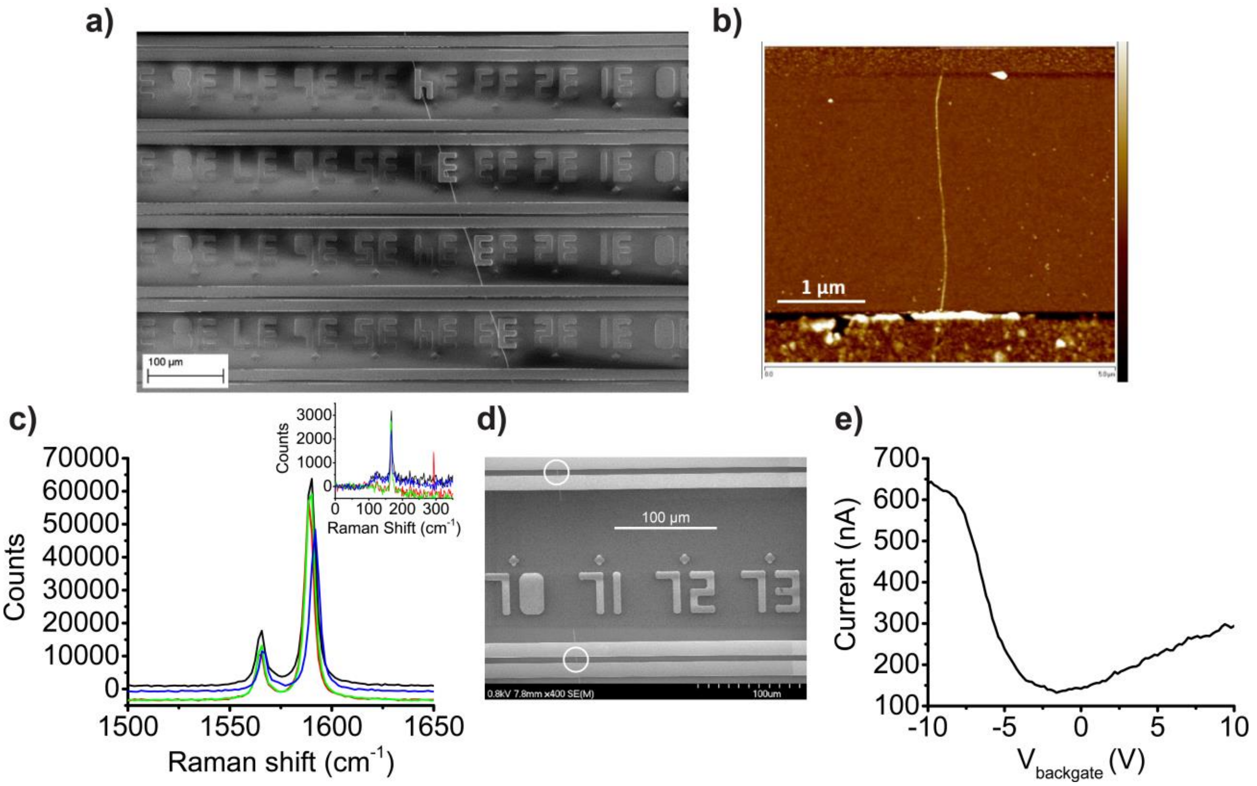

Supplementary Figure 2. CNT FET devices are extensively characterized. a) SEM image showing a CNT spanning five electrode pairs. b) AFM image of a CNT fragment capped by a source and drain Titanium electrodes. c) Raman spectra collected from individual CNT fragments isolated from the same CNT shown in a, demonstrating reproducible Raman features, with ω_G^+ and ω_G^- located at 1588 cm^{-1} and 1566 cm^{-1} ,respectively. Inset shows the RBM spectra of the same nanotubes, located at 170 cm^{-1} corresponding to a diameter of 1.45nm[11]. One of the fragments contains an additional peak at 295 cm^{-1} possibly corresponding to an inner wall or an additional fragment (bundle) with diameter of 0.84nm. d) SEM image showing two CNT fragments after O_2 plasma isolation. e) I-V curve of a CNT device backgated in air showing an ambipolar transport characteristics.

Supplementary Note 3

Nano confined chemistry and FBDP coupling

E-beam nanolithography (Nanobeam, UK) generates an exposed area of: 30 nm × 24 µm in an ultrathin (30-nm-thick) PMMA resist layer. These “nanowells”, subsequently used to confine the reaction chemistry, are characterized by AFM, as shown in Supplementary Figure 3a. Reaction with the diazonium salt FBDP, shown in Figure S3b, is confined to a 30 nm exposed CNT sidewall.

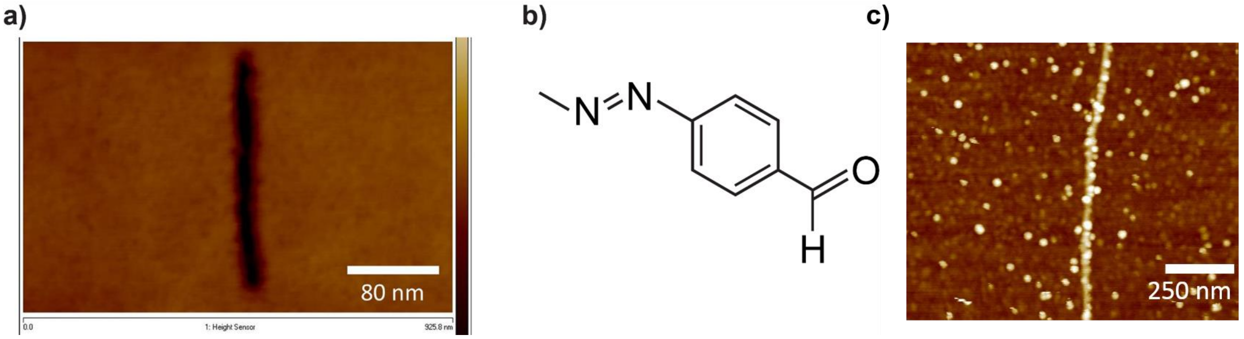

Supplementary Figure 3. A nanoconfined reaction of a CNT with the diazonium salt FBDP. a) AFM image showing an exposed 30 nm “nanowell” in a PMMA resist following e-beam lithography and development, confining a small area over the CNT sidewall to control the diazonium reaction chemistry. b) FBDP molecule. c) Specific CNT coupling of FBDP is indicated by labeling with amine-modified gold nanoparticles (in bulk). Minimal background adsorption is observed.

In our previous studies of nano confined chemistry[12], we have shown that a physically nanoconfined well allows for only a small number of potential adducts – due to the combined effect of diffusion limitation, reaction chamber dimensions, and steric effects. A calculated average of ~1.4 functional groups per device, which corresponds to a conductance drop of < 20% was measured in I-V probing. Nanowell dimensions above 30 nM resulted in significantly higher conductance drops. Furthermore, even if few defect sites were introduced, their proximity would not allow more than one oligonucleotide to bind to a functionalized site. Also, the yield associated with this DNA functionalization step help to ensure either single-molecule or no attachment to a smFET device (the reductive amination reaction in itself is not highly efficient when the concentration is so low).

Non-specific adsorption or grafting of FBDP to the surrounding SiO_2 surface was shown to be minimal, as evident from amine-modified gold nanoparticles labeling, seen in Supplementary Figure 3c (reaction with FBDP was carried out in bulk with an incubation time of 1 hour in 0.1 M PB followed by reductive amination as previously described). Specific attachment to the CNT is visible, in comparison with the background.

Supplementary Figure 4

Experimental SiO_2 chip

The chip was fabricated, as described in the Methods, resulting in an array of 58 electrode pairs and two platinum pseudo-reference electrodes. An illustration of the chip with the PDMS microfluidic cell and an image of the chip are shown in Supplementary Figures 4a, b. An image of the custom made printed circuit board is shown in Supplementary Figure 4c.

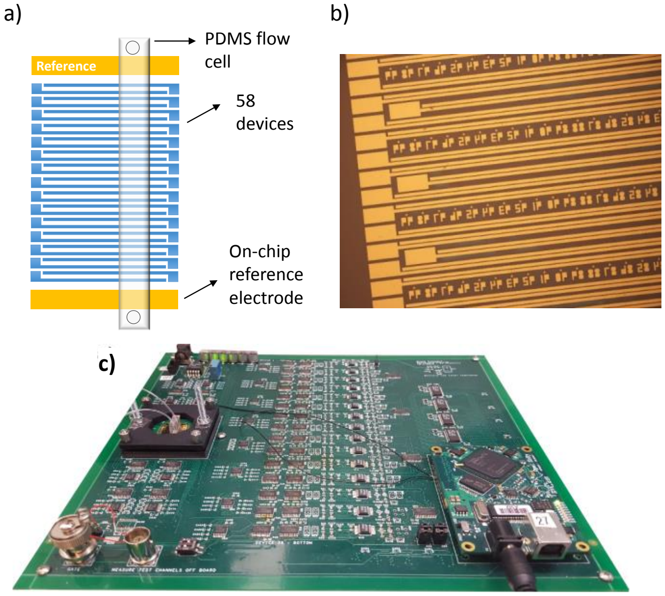

Supplementary Figure 4. Experimental passive chip and supporting electronics. a) Scheme showing the electrode configuration with 58 pairs of Titanium source and drain electrodes and two Platinum pseudo-reference electrodes. A PDMS flow cell is stamped over the isolated CNT devices. b) Microscope image of a fully fabricated chip. c) Photograph of the custom made printed circuit board setup used for electronic measurements.

Supplementary Note 5

Real-time conductance measurements for temperature-dependent controls

The RTN-like behavior that is observed for the trajectories in figure 2a is attributed to carrier scattering from localized energy states[13] at the point of attachment that is modulated by charge within a few Debye lengths (typically 3 nm or so) of the point of attachment[14]. Anionic charges generally reduce smFET resistance by creating a lower resistance path for holes.

Following DNA probe coupling, the chips were extensively rinsed and real-time conductance measurements were recorded to confirm that no RTN-like signal was observed. An example for this control is shown in Supplementary Figure 5a. Additionally, a non-complementary target ssDNA (a similar length 20-mer oligonucleotide with the sequence: 5’ GTGATTTCACTTGCAATGTC) was measured at the same temperatures and concentrations to confirm that activity is not caused by non-specific adsorption or by a doping mechanism. An example for this control is shown in Supplementary Figure 5b. Prior to introduction of the different oligonucleotide targets, the control measurement of a tethered probe (entitled ‘probe only’) shows a stable signal characterized by 1/f noise with no observed RTN. The CNT transport characteristics are clearly visible. While conductance levels are at ~ 650 nA for a device biased at V_g=-300mV and V_g=0V, once the gate is changed to +300mV, the conductance drops to ~450nA. The non-complementary ssDNA control looks very similar with no observed RTN activity.

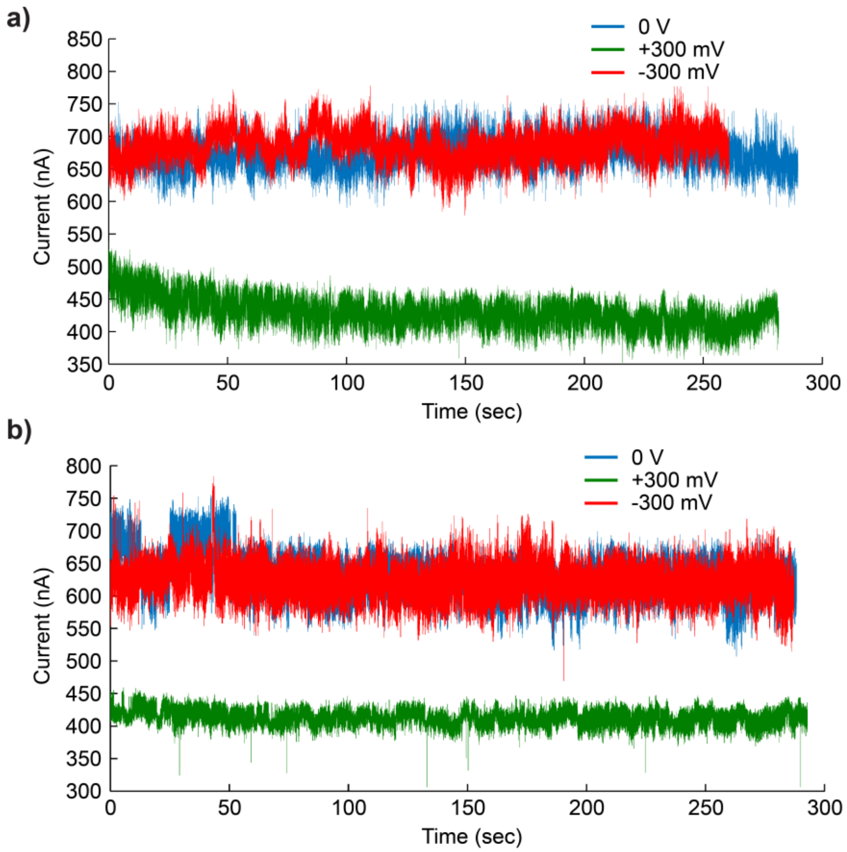

Supplementary Figure 5. Control time traces for the temperature-dependent melting experiments. a) smFET conductance measurements (V_{SD}=100mV) of a CNT device with a tethered ssDNA probe in 43 mM Phosphate buffer at 50 °C. RTS is not observed over a 280 sec period. Conductance drops at a Vg=+300mV reflecting the device *I-V* characteristics. b) Control measurement of a functional CNT device following the addition of a 100 nM fully non-complementary 20-mer oligonucleotide (at 50 °C). RTS activity is not observed over a 290 sec period. Conductance levels remain similar to before.

Supplementary Figure 6.

Survival probability plots for temperature-dependent data

Survival probability plots for 30 °C, 40 °C, and 60 °C are shown in Supplementary Figure 6. A double exponential fit is used to extract hybridization and melting rate constants.

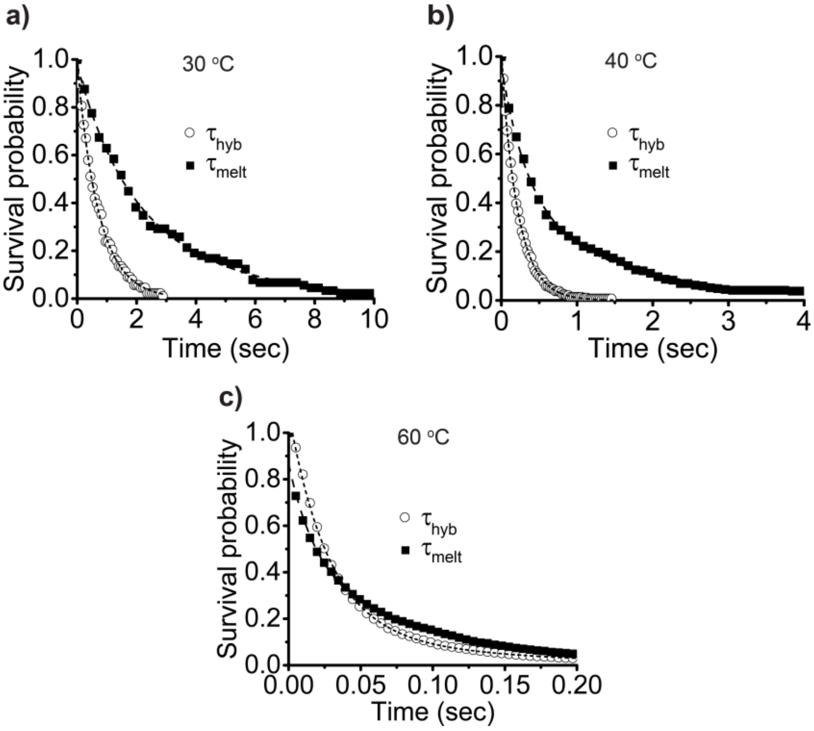

Supplementary Figure 6. Reaction rate constants are extracted from fits to survival probability plots. Cumulative density functions normalized to the maximum event count depict events life time distribution (survival probability) for a reaction with 100 nM complementary 20-mer ssDNA at a) 30 °C, b) 40 °C and c) 60 °C.

Supplementary Note 7

Hybridization-mode rate constants

Reaction kinetic rates are extracted by fitting the exponential decay of the survival probability in each conductance state. Because of the double exponential decay of each survival plot, the hybridization reaction has two rate constants, as previously described, which are associated with diffusion. The hybridization reaction consists of two modes, a 1D surface-based reaction and a bulk 3D hybridization reaction. 1D diffusion is expected to be faster due to the reduction-of-dimensionality (RD enhancement), which has been

previously validated in biochemical systems[15]. At low target concentrations the 1D hybridization rate is thus accelerated by high surface diffusion coefficients, while for high target concentrations, the 1D rate is considerably decreased due to a much slower diffusion (1D diffusion coefficients are inversely proportional to concentration[16, 17, 18]). This is clearly shown by the 1D hybridization rates plotted in Supplementary Figure 7a ($k_{hyb,fast}$ for 1 nM and 10 nM and $k_{hyb,slow}$ for 100 nM). The reaction on the surface is diffusion-limited for the 100 nM but reaction-limited for the 1 and 10 nM. The 3D mode, on the other hand, is a diffusion-limited reaction at low concentrations (1 and 10 nM) and reaction-limited at 100 nM. This is shown in Supplementary Figure 7b ($k_{hyb,slow}$ for 1 nM and 10 nM and $k_{hyb,fast}$ for 100 nM).

This transition point between rate-determining steps, occurring at a concentration of 100 nM, has been previously reported[19]. The 3D hybridization rates show concentration dependence from 1 nM to 10 nM, as expected. At 100 nM the 3D rates do not significantly increase since they are reaction limited. The 1D diffusion of the 100 nM, on the other hand, is limited by steric interferences caused by other 20-mers that are likewise adsorbed to the surface. In this case, a repulsive electrostatic force exerted by bias alleviates these steric effects, as shown in Supplementary Figure 7c. upon the application of 300 mV bias the 1D hybridization of the 100 nM is significantly increased. 1D hybridization rates at lower concentrations are not affected by this bias since they are not impeded by these steric effects.

It should be noted that these two hybridization modes are not completely de-convolved by taking the low and fast time constants from double-exponential fits. At low target concentrations, the slow rate is primarily the 3D mode but is still influenced by surface binding. Similarly, for higher concentration, the fast rate representing the 3D mode is likely to be influenced by 1D hybridization events.

The melting rates (with a choice of slow and fast time constants that matches that used for hybridization) show no dependence on concentration but rather a strong temperature dependence (as shown in Supplementary Figure 7d).

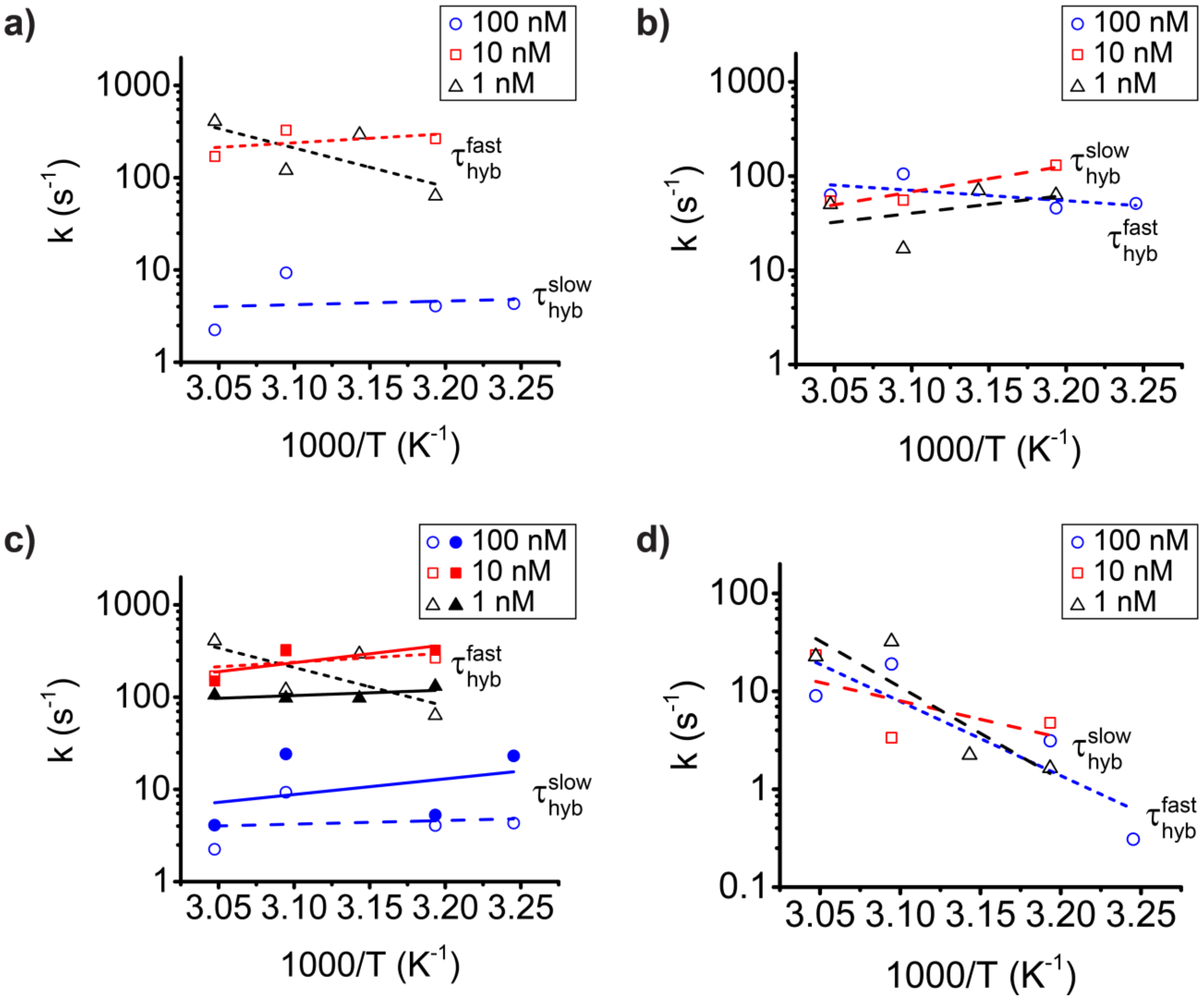

Supplementary Figure 7. Surface versus solution hybridization and melting modes. a) Surface-based (1D) hybridization as a function of temperature, characterized by τ_{hyb}^{slow} for 100 nM (blue circles) and τ_{hyb}^{fast} for 10 nM (red squares) and 1 nM (black triangles). The rates are comparable for 1 and 10 nM concentrations, but reduced for 100 nM. b) Solution-based (3D) hybridization as a function of temperature, characterized by τ_{hyb}^{fast} for 100 nM (blue circles) and τ_{hyb}^{slow} for 10 nM (red squares) and 1 nM (black triangles). c) Surface-based hybridization, as a function of temperature, with and without bias. Open shapes and dashed lines indicate rates at $V_g = 0$ V, and filled shapes and solid lines designate rates at $V_g = 300$ mV. With applied bias, rates for 100 nM are increased, but are marginally affected for 1 and 10 nM. d) Solution-based melting rates as a function of temperature, at $V_g = 0$ V. Melting rates are not concentration-dependent.

Supplementary Note 8

Real-time conductance measurements - temperature dependent melting of 100 nM complementary target

At the calculated melting temperature of 50 °C for the complementary target, the dwell time in each state is expected to be equal. A 10-sec time trace is presented in Supplementary Figure 8 along with a current histogram showing that at 50 °C both states are nearly equal.

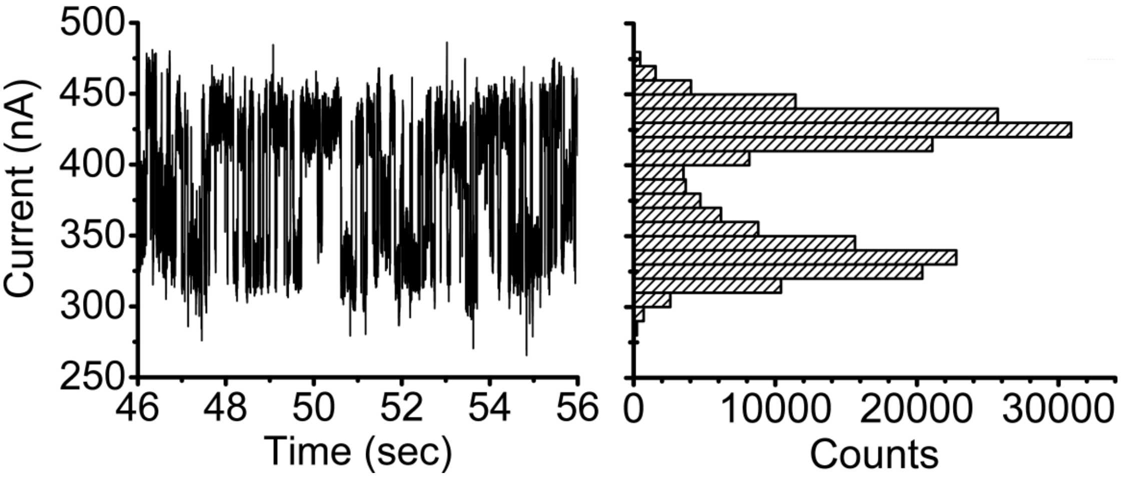

Supplementary Figure 8: Two state conductance affected by temperature-dependent DNA melting. A 10 sec time trace of a 100 nM complementary ssDNA at 43 mM phosphate buffer and 50 °C demonstrates RTS with distinct two states and a nearly equal time spent in each conductance state corresponding to a hybridized fraction of 0.5, as expected at the T_m.

Supplementary Figure 9

Bias dependent DNA hybridization

Supplementary Figure 9 shows current histograms for the data in Figure 4a.

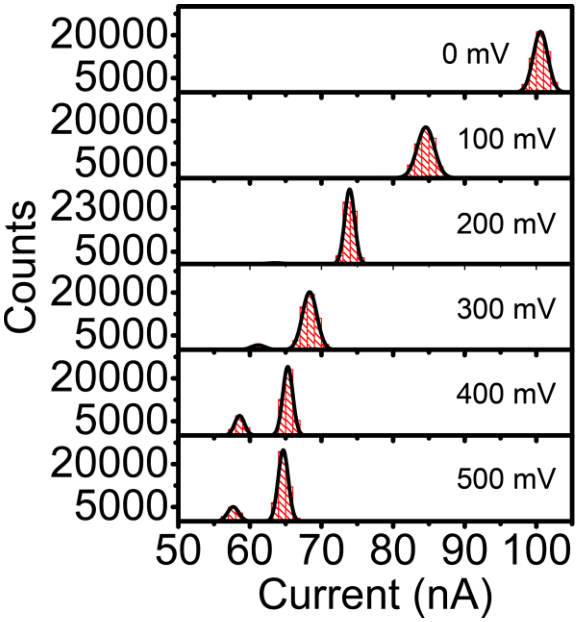

Supplementary **Figure 9.** 'E-melting' of DNA at low temperature. Once a complementary ssDNA is introduced, two-state activity, attributed to melting and hybridization, is not observed unless a potential is applied. With increasing potential, melting events, reflected by a lower conductance state, become longer and more frequent**.**

<u>Supplementary Figure 10</u>

Control traces (non-complementary DNA) for bias-dependent melting data

At a fixed temperature of 40 °C and with 100 nM of non-complementary DNA floating in solution, the device does not exhibit bi-stable RTS signals at any positive solution bias as shown in Supplementary Figure 10.

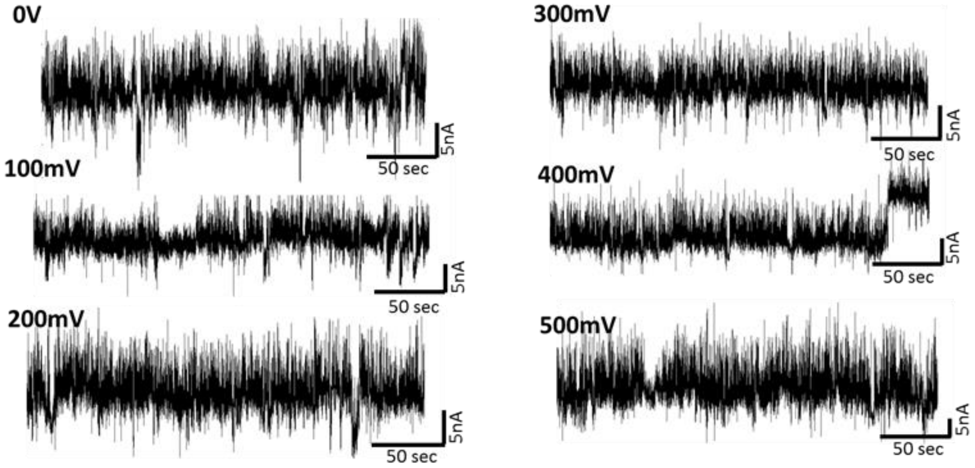

Supplementary Figure 10. A control real-time conductance measurement. At 40 °C RTS is not observed when a non-complementary target is introduced, further supporting the specificity of the signal, and the feasibility of the smFET device.

Supplementary Figure 11

Effect on SNR of the application of a solution gate bias

Supplementary Figure 11 shows the dependence of SNR on gate bias.

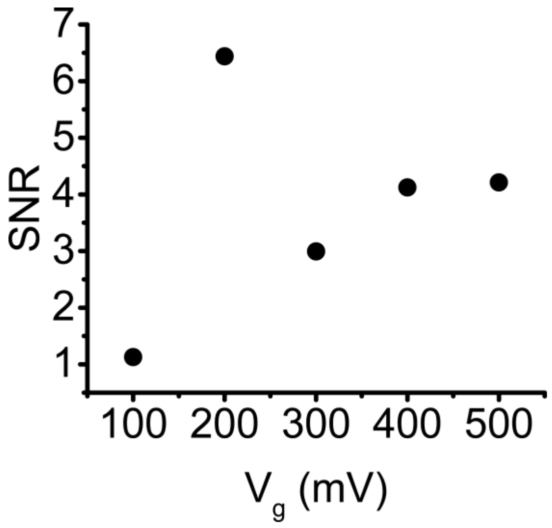

Supplementary Figure 11. Even the FET device performance is expected to change with solution bias modulation, there is also a general trend toward higher signal-to-noise ratio (SNR) with increased bias. This observation signifies that we are sensing a single-molecule and not just purely changing device characteristics. The SNR is calculated by the difference between the histogram peaks (ΔI) divided by the full-width half maximum (FWHM) of the upper peak.

Supplementary References

1. Bahr JL, Yang JP, Kosynkin DV, Bronikowski MJ, Smalley RE, Tour JM. Functionalization of carbon nanotubes by electrochemical reduction of aryl diazonium salts: A bucky paper electrode. *J Am Chem Soc* **123**, 6536-6542 (2001).

2. Dyke CA, Stewart MP, Maya F, Tour JM. Diazonium-based functionalization of carbon nanotubes: XPS and GC-MS analysis and mechanistic implications. *Synlett*, 155-160 (2004).

3. Strano MS, *et al.* Electronic structure control of single-walled carbon nanotube functionalization. *Science* **301**, 1519-1522 (2003).

4. Bahr JL, Tour JM. Highly functionalized carbon nanotubes using in situ generated diazonium compounds. *Chem Mater* **13**, 3823-+ (2001).

5. Nair N, Kim WJ, Usrey ML, Strano MS. A structure-reactivity relationship for single walled carbon nanotubes reacting with 4-hydroxybenzene diazonium salt. *J Am Chem Soc* **129**, 3946-3954 (2007).

6. Gavrilyuk J, Ban H, Nagano M, Hakamata W, Barbas CF. Formylbenzene Diazonium Hexafluorophosphate Reagent for Tyrosine-Selective Modification of Proteins and the Introduction of a Bioorthogonal Aldehyde. *Bioconjugate Chem* **23**, 2321-2328 (2012).

7. Haque AJ, Kim K. Aldehyde-Functionalized Benzenediazonium Cation for Multiprobe Immobilization on Microelectrode Array Surfaces. *Langmuir* **27**, 882-886 (2011).

8. Hermanson GT. Bioconjugate Techniques, 2nd Edition. *Bioconjugate Techniques, 2nd Edition*, 1-1202 (2008).

9. Dresselhaus MS, Dresselhaus G, Saito R, Jorio A. Raman Spectroscopy of Carbon Nanotubes. *Cont Concept Condens*, 83-108 (2008).

10. McEuen PL, Fuhrer MS, Park HK. Single-walled carbon nanotube electronics. *Ieee T Nanotechnol* **1**, 78-85 (2002).

11. Araujo PT, Pesce PBC, Dresselhaus MS, Sato K, Saito R, Jorio A. Resonance Raman spectroscopy of the radial breathing modes in carbon nanotubes. *Physica E* **42**, 1251-1261 (2010).

12. Bouilly D, *et al.* Single-Molecule Reaction Chemistry in Patterned Nanowells. *Nano Lett* **16**, 4679-4685 (2016).

13. Prisbrey L, Roundy D, Blank K, Fifield LS, Minot ED. Electrical Characteristics of Carbon Nanotube Devices Prepared with Single Oxidative Point Defects. *J Phys Chem C* **116**, 1961-1965 (2012).

14. Sharf T, *et al.* Single electron charge sensitivity of liquid-gated carbon nanotube transistors. *Nano Lett* **14**, 4925-4930 (2014).

15. Axelrod D, Wang MD. Reduction-of-dimensionality kinetics at reaction-limited cell surface receptors. *Biophys J* **66**, 588-600 (1994).

16. Chan V, Graves DJ, Fortina P, McKenzie SE. Adsorption and surface diffusion of DNA oligonucleotides at liquid/solid interfaces. *Langmuir* **13**, 320-329 (1997).

17. Chan V, Graves DJ, McKenzie SE. The biophysics of DNA hybridization with immobilized oligonucleotide probes. *Biophys J* **69**, 2243-2255 (1995).

18. Chan V, McKenzie SE, Surrey S, Fortina P, Graves DJ. Effect of hydrophobicity and electrostatics on adsorption and surface diffusion of DNA oligonucleotides at liquid/solid interfaces. *Journal of Colloid and Interface Science* **203**, 197-207 (1998).

19. Erickson D, Li D, Krull UJ. Modeling of DNA hybridization kinetics for spatially resolved biochips. *Anal Biochem* **317**, 186-200 (2003).
